# Belowground bud banks and land use change: roles of vegetation and soil properties in mediating the composition of bud banks in different ecosystems

**DOI:** 10.3389/fpls.2023.1330664

**Published:** 2024-01-05

**Authors:** Jing Wu, Xianzhang Hou, Lan Xu, Quanlai Zhou, Yongcui Wang, Ziwu Guo, Michael Opoku Adomako, Qun Ma

**Affiliations:** ^1^ Zhejiang Provincial Key Laboratory of Plant Evolutionary Ecology and Conservation, School of Life Sciences, Taizhou University, Taizhou, China; ^2^ Department of Natural Resource Management, South Dakota State University, Brookings, SD, United States; ^3^ Institute of Applied Ecology, Chinese Academy of Sciences, Shenyang, China; ^4^ Research Institute of Subtropical Forestry, Chinese Academy of Forestry, Hanzhou, China; ^5^ Institute of Wetland Ecology and Clone Ecology, Taizhou University, Taizhou, China

**Keywords:** bud density, clonal organs, land use change, storage organs, vegetation density

## Abstract

**Introduction:**

Belowground bud banks play integral roles in vegetation regeneration and ecological succession of plant communities; however, human-caused changes in land use severely threaten their resilience and regrowth. Although vegetation attributes and soil properties mediate such anthropogenic effects, their influence on bud bank size and composition and its regulatory mechanisms under land use change have not been explored.

**Methods:**

We conducted a field investigation to examine impacts of land use change on bud bank size and composition, vegetation attributes, and soil properties in wetlands (WL), farmlands (FL), and alpine meadow (AM) ecosystems in Zhejiang Province, China.

**Results:**

Overall, 63 soil samples in close proximity to the vegetation quadrats were excavated using a shovel, and samples of the excavated soil were placed in plastic bags for onward laboratory soil analysis. The total bud density (1514.727 ± 296.666) and tiller bud density (1229.090 ± 279.002) in wetland ecosystems were significantly higher than in farmland and alpine meadow ecosystems [i.e., total (149.333 ± 21.490 and 573.647 ± 91.518) and tiller bud density (24.666 ± 8.504 and 204.235 ± 50.550), respectively]. While vegetation attributes critically affected bud banks in WL ecosystems, soil properties strongly influenced bud banks in farmland and alpine meadow ecosystems. In wetland ecosystems, total and tiller buds were predominantly dependent on soil properties, but vegetation density played a significant role in farmlands and alpine meadow ecosystems. Root sprouting and rhizome buds significantly correlated with total C in the top 0 – 10 cm layer of farmland and alpine meadow ecosystems, respectively, and depended mainly on soil properties.

**Discussion:**

Our results demonstrate that land use change alters bud bank size and composition; however, such responses differed among bud types in wetland, farmland, and alpine meadow ecosystems.

## Introduction

Human-derived modifications of terrestrial ecosystems, including land use changes, underlie the altered belowground bud bank densities, vegetation regeneration, and ecological succession, leading to global biodiversity loss and ecosystem services (Allan et al., 2015; Newbold et al., 2015; Chang and Turner, 2019; Winkler et al., 2021; Simkin et al., 2022). Agricultural activities (e.g., pesticides and herbicide application) and expansion of arable croplands have predominantly shifted many crucial attributes of the natural vegetation (Zhao et al., 2023), with substantial implications on belowground bud banks (storage organs e.g., rhizomes, corm, and ramets) that are vital for vegetation regrowth, aboveground recruitment, productivity (Li et al., 2018; Qian et al., 2021; Hou et al., 2022). Although expanding the arable cropland greatly affects many plant species and bud banks, their responses may differ across ecosystems due to differences in the vegetation cover and soil characteristics that support plant growth (Newbold et al., 2015; Semenchuk et al., 2022). Likewise, the land use intensity and impacts on belowground bud banks may also differ between such ecosystems. Despite these differential responses to land use change, the role of vegetation and soil attributes in mediating bud bank responses in different ecosystems has not been explored. In light of the ongoing environmental change on a worldwide scale, it is imperative to comprehend the role of vegetation and soil characteristics to develop appropriate management techniques that will increase their ecological relevance.

Soil characteristics (e.g., moisture, nutrients, and particle sizes) constitute an essential component driving the structure and vegetation cover, as well as nutrient availability of terrestrial ecosystems (Wu et al., 2020; Adomako et al., 2021; Inoue et al., 2022), and play critical roles in regeneration, growth, and productivity of plants (Zuo et al., 2009; Hoover et al., 2014). Soil properties are important ecological parameters that determine the magnitude, distribution pattern, and vegetation succession, which are a function of soil aggregate particles, belowground bud bank density, and resprouting ability (Clarke et al., 2013; Wu et al., 2020). For example, in a soil substrate heterogeneity study, Adomako et al. (2021) reported that a ceramsite-quartz mixture with larger aggregate particle sizes significantly decreased ramets growth of *Leymus chinensis* compared to plants grown in field soil with relatively smaller particle sizes, as the former substrates have greater mechanical resistance to seed or vegetative sprouting than the latter substrates (Semchenko et al., 2008). Moreover, higher clay and low nutrient content in wetland soils may decrease resprouting and growth of tiller buds than resprouting and growth of tillers in farmlands and alpine meadows (Reddy et al., 2013; Mobilian and Craft, 2021). The soil physicochemical properties strongly influence the aboveground vegetation recruitment from seeds or belowground bud banks (Adomako et al., 2021; Inoue et al., 2022; Wu and Yu, 2022). Such driving force underpins aboveground vegetation recruitment dynamics, vegetation density, and productivity of plant species in natural ecosystems (Peng et al., 2015; Adomako et al., 2021). However, information regarding interaction effects of land use change and soil characteristics on bud banks across varying ecosystem types is limited in our current understanding.

In natural systems, belowground bud banks play a pivotal role in maintaining plant biodiversity against anthropogenic and natural disturbances, such as farming and climate change (Plue and Cousins, 2018; Ott et al., 2019). Particularly in vegetations dominated by perennial species, belowground bud banks serve as ecological insurance against short- and long-term disturbances such as drought, wildfire, and grazing (Deng et al., 2014; Hoover et al., 2014; Ott et al., 2019). Although long-term effects of some ecological perturbations can adversely affect bud bank density (VanderWeide and Hartnett, 2015; Qian et al., 2023), they may likely differ between ecosystems owing to differences in vegetation cover, species composition, and soil characteristics. These differential responses of bud banks across varying ecosystems may explain variations in resilience and resprouting capacity after disturbances (Xu et al., 2021). For instance, plants adapted to grow in drier conditions (i.e., alpine meadow in our study) showed strong resistance to chronic drought stress compared to wetland plants growing under the same stressful condition (Luo et al., 2023; Ren et al., 2023). Likewise, the abundance and density of belowground organs (e.g., rhizomes and tillers) of species may also differ in their responses to disturbance in their respective ecosystems (Xu et al., 2021; Qian et al., 2022; Klimešová et al., 2023). Despite the critical roles of belowground bud banks to plant community stability and productivity, how vegetation and soil characteristics mediate their responses to land-use changes are poorly understood.

Bud types (e.g., rhizome, tiller, and root sprouting buds) play different functional roles and determine diverse adaptive strategies under variable ecosystems (Zhang et al., 2019). Belowground bud banks are widely sourced from rhizome and tiller buds (Zhang et al., 2009; Ott and Hartnett, 2015). In addition to adventitious root buds, tiller buds derived from the base of the parent shoots for hemicryptophyte, rhizome buds, and root sprouting buds mainly initiate from underground roots and rhizomes for geophyte. Previous studies reported that tiller buds are more closely related to vegetation attributes, while rhizome and root sprouting buds are sensitive to water or nutrient concentrations in surrounding habitats (Passioura, 1988). However, how vegetation attributes and soil properties interact to determine the responses of different bud bank types under the ongoing land use changes is unknown.

To explore the changes in bud bank size and composition and its regulatory mechanism under land use change, we selected two plots in Linhai and Taizhou sampling sites for wetlands (WL), farmlands (FL), and alpine meadows (AM), respectively. We took 63 sampling points in total for the bud demographic. We also measured biotic (vegetation density, aboveground biomass, and Shannon-Weiner diversity index) and abiotic parameters (soil moisture content, total carbon (C), and total N) relevant to the bud bank density for different plant functional groups. Specifically, we aim to explore (1) changes in/patterns of the bud bank traits among different land use types and (2) the role of vegetation attributes and soil properties in determining bud demographic and bud densities of different bud bank types under land use change.

## Materials and methods

### Study sites

**Figure 1:**
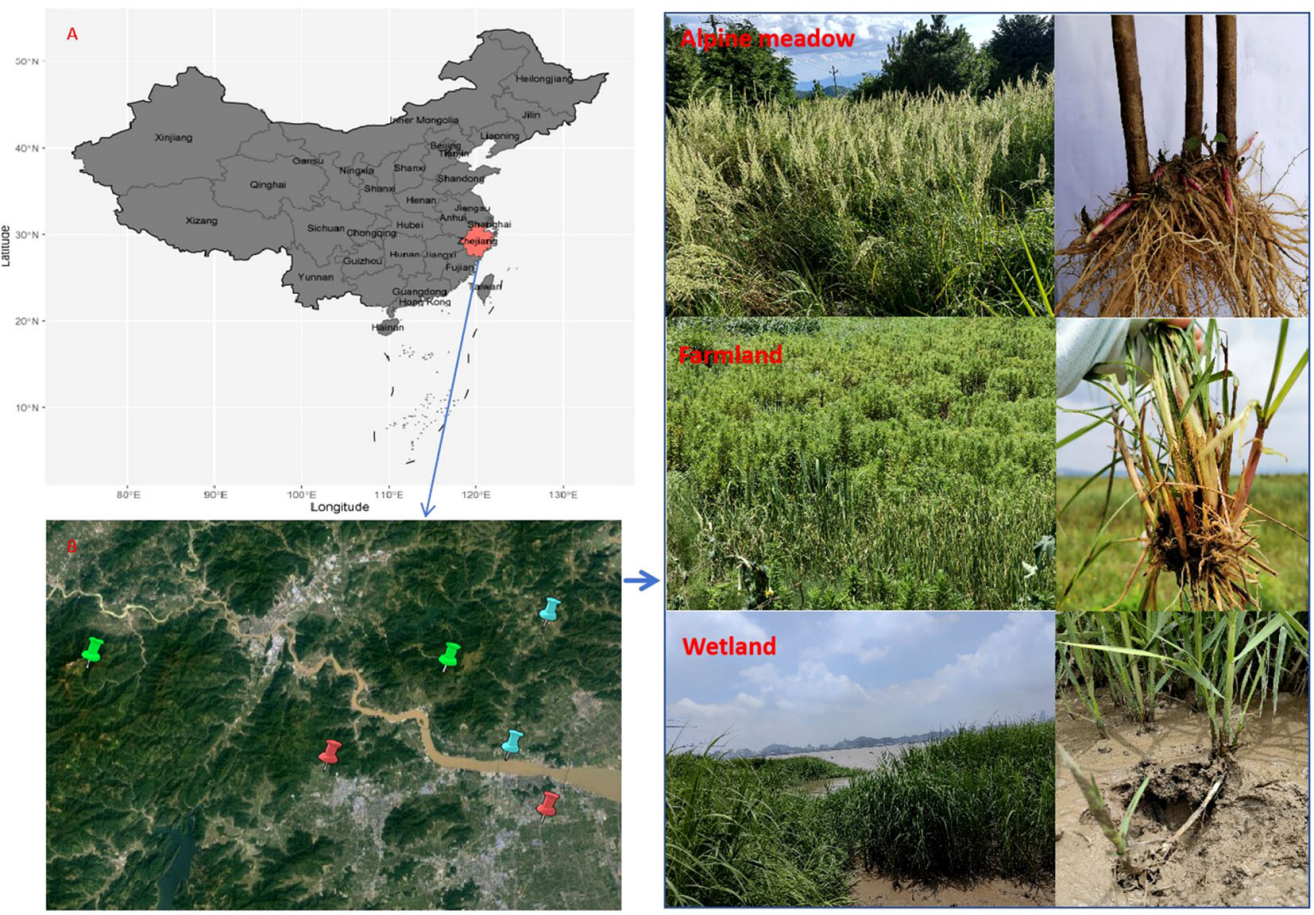
Location of Zhejiang Province **(A)** and the six sampling sites **(B)**, including two sites for farmland (red label) in Taizhou City, two sites for Alpine meadow (green label) in Linhai City, one site for wetland (blue label) in Taizhou city and one site for wetland (blue label) in Linhai city, respectively.

The study was conducted in Linhai and Taizhou City (120°17′-121°56′E, 28°01′-29°20″N), southeastern Zhejiang Province, China (**Figure 1A**). In response to rapid modern development and urbanization of China, Linhai and Taizhou Cities located along the coast are undergoing massive expansion of industries and settlement, cascading potential impacts on its vegetation structure and succession. Therefore, we conducted this field study to examine how the rapid land use change may influence belowground bud banks to highlight the long-term effects on vegetation structure and dynamics in this area and beyond. The region has a typical subtropical monsoon climate with moderate annual temperatures, abundant sunshine, and precipitation; thus, the growing season lasts from late April to late September before the yearly winter commences. The landscape consists of a mosaic of forests, arable lands, and wetlands. Six sample sites in total were selected in Linhai and Taizhou City: two alpine meadows sites located in Kuocang Mountain and Lantian Mountain, two farmland sites located next to residential quarters at the foot of mountain, and two wetland sites situated next to Xiaozhi reservoir and Jiaojiang river, respectively (**Figure 1B**). The dominant vegetation at the wetland site comprises Phragmites communis, Arundo donax, Imperata cylindrica, Solidago canadensis, and other herbaceous plants. Some perennial and annual herbs, such as Juncus effusus, Imperata cylindrica, Lysimachia fortune, and Rubus phoenicolosius, dominate the alpine meadows’ vegetation. The vegetation in WL, FL, and AM ecosystems experience the same climatic conditions, i.e., similar rainfall patterns, temperature, and humidity.

### Field observations and bud bank sampling

In August 2022, we selected two plots for WL, FL, and AM in each sampling site and established transects 10 m apart between each chosen plot. In each plot, 22 sampling points were selected for wetlands, 24 for farmlands, and 17 for alpine meadows, making 63 sampling points in total. In each sampling point, 1 × 1 m quadrats were established to record the vegetation composition, identify all species within each community, and record the number of each species identified and their abundance, as well as their average height and plant density. With grasses and sedges, we counted and recorded the number of ramets (e.g., *Phragmites communis*) and calculated the number of individuals for discrete species. We used the number of all ramets/individuals/m^2^ in all quadrats to estimate the vegetation density. Additionally, we sampled soils between the top 0 – 10 cm to measure soil water and nutrient content from each ecosystem using a ring cutter and soil drill with a diameter of 7 cm.

We dug 25 × 25 × 25 cm quadrats in each vegetation quadrant to record the belowground bud bank composition at each sampling point, totaling 63 quadrats in the three ecosystems. All samples were processed within two weeks, and no rotting was observed during this period. Only turgid bud tissue was counted, and tissues with necrotic signs or visibly dead tissues were discarded. We defined three types of bud banks according to the morphological characteristics of bud-bearing organs: rhizome buds (axillary buds and apical buds on hypogeogenous rhizomes), tiller buds (axillary buds at the shoot bases of caespitose species and rhizomatous grasses); and root-sprouting bud (adventitious bud formed mainly endogenously on roots of forb or shrub). In contrast to counting buds directly on rhizomes, stems, and roots, dissecting those at the base of shoots was necessary to estimate the number of tillers and root buds.

### Biomass and diversity calculation

The aboveground biomass per quadrat was measured by clipping the plants at ground level. The respective biomass was dried at 70°C to constant weight. We measured species diversity indices for each quadrat using the following equations (Li et al., 2014):


Importance value (IV) = (relative height + relative density)/2

$$\text{Shannon-Wiener Index }\left(\text{H}\right)\text{ = - }\sum \left(P_{i}\;\text{ln}\;P_{i}\right)\;$$

*P_i_* is the relative importance value of the i_th_ species in the community.

### Data analysis

The original dataset of bud densities was converted into numbers of buds per square meter. The average bud density of each sampling position was then calculated for further analysis. One-way ANOVA was applied to analyze differences in belowground bud bank density, vegetation characteristics (vegetation density, aboveground biomass, and Shannon-Weiner diversity index), and soil properties (soil water content, total C, total N) among WL, FL, and AM ecosystems. One-way ANOVA and Tukey’s honestly significant difference *post hoc* test were performed using SPSS 18.0 (SPSS Inc., USA). Redundancy Analysis (RDA) was used to examine the correlations between belowground bud bank and aboveground vegetation, soil, and properties in the WL, FL, and AM ecosystems. Aboveground vegetation information included vegetation density, Shannon-Weiner diversity index, and aboveground biomass, soil properties embraced soil water content of 0 − 10 cm layer, total C of 0−10 cm layer, and total N of 0−10 cm layer. The original data was log-transformed and normalized before RDA. Some environmental factors were deleted by Monte Carlo selection under *P< 0.05.* We selected variables with high canonical loading factors, confirmed by a cutoff value of 0.35, and parameters highly correlated with canonical variables detected by high standardized coefficients (r > 0.4). RDA was performed using CANOCO *v. 4.5*.

## Results

### Change in bud bank traits among different land use types

**Figure 2:**
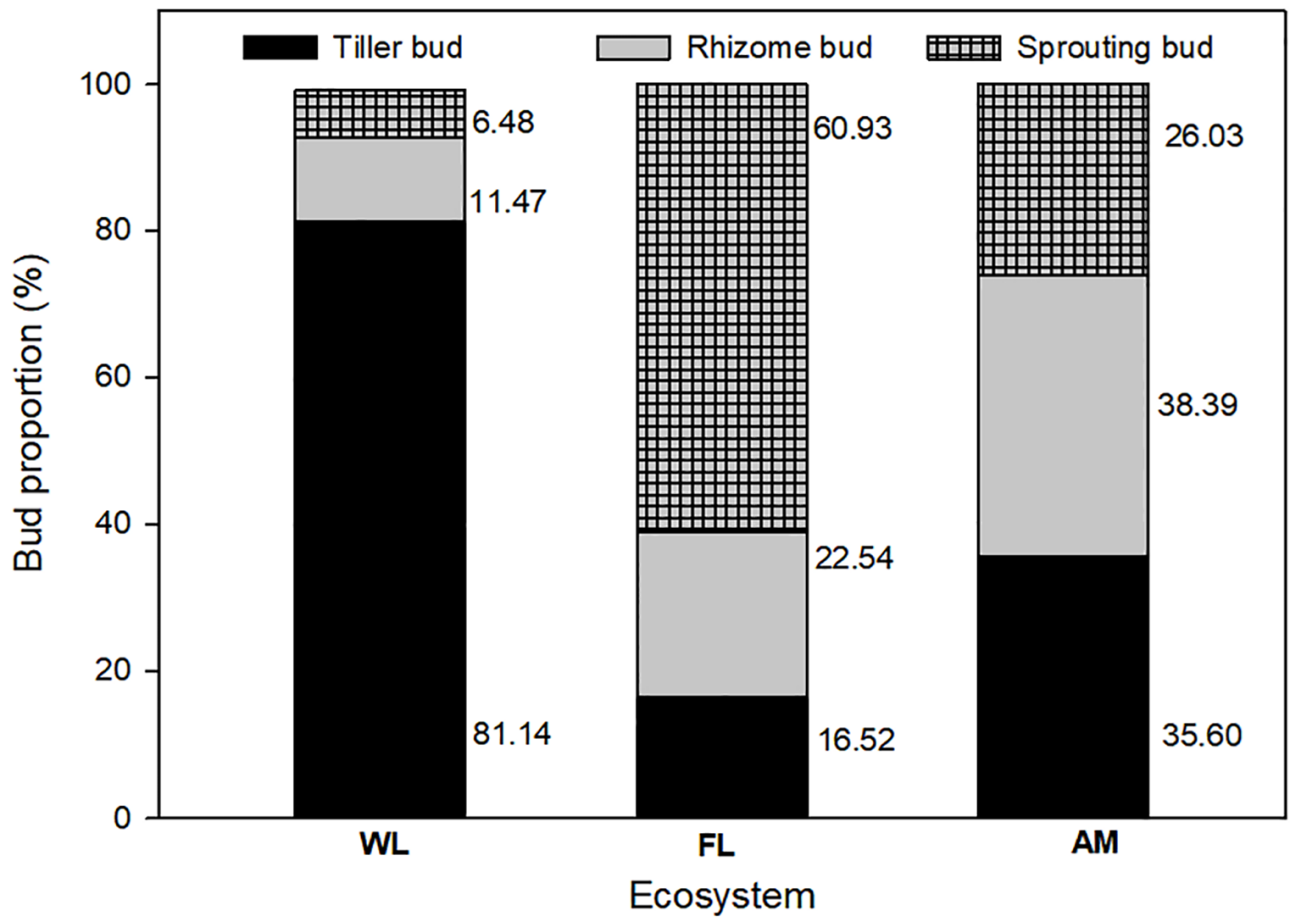
Percentage of belowground bud bank density of different bud types in wetland (WL), farmland (FL), and Alpine meadow (AM) ecosystems.

**Figure 3:**
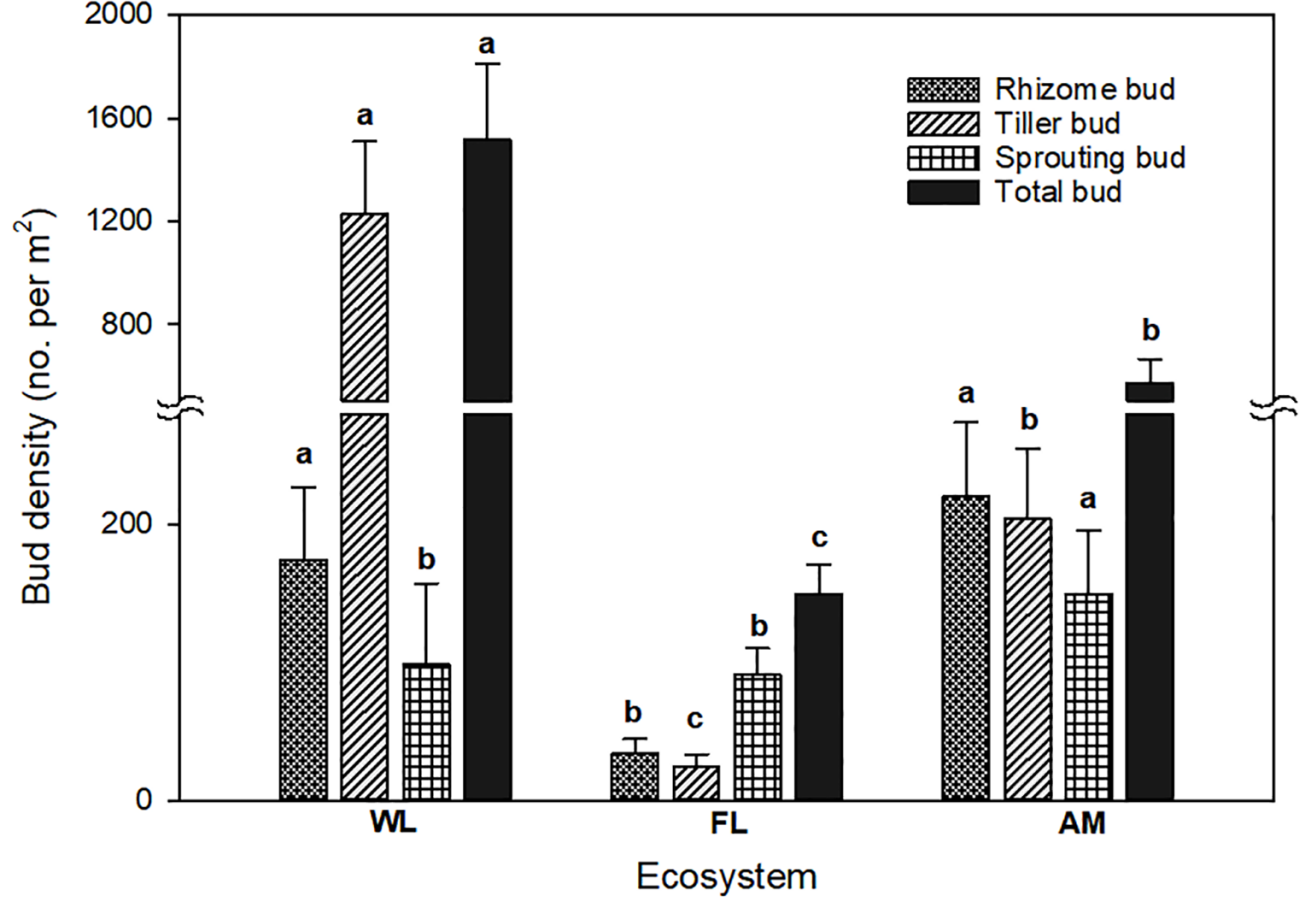
Difference in bud density of different bud types in WL, FL, and AM ecosystems. Lowercase letters indicate significant differences (*P< 0.05*) in bud density among land use types. Bars represent error bars.

We found rhizome, tiller, and root sprouting buds in WL, FL, and AM ecosystems; however, land use change affected the bud bank structure, with tiller buds accounting for the majority (81.14%) of total buds in WL ecosystems and sprouting buds (60.93%) showed dominance in FL ecosystems. In contrast, rhizome buds accounted for the highest proportion in the AM ecosystem (**Figure 2**). Bud bank density and soil properties differed significantly among WL, FL, and AM ecosystems (**Table 1**). The total bud density (1514.727 ± 296.666) and tiller bud density (1229.090 ± 279.002) in WL were significantly higher than that in FL and AM (*P< 0.01*, **Figure 3**). Additionally, rhizome bud density (220.235 ± 53.516) and root sprouting bud density (149.294 ± 46.496) in AM were significantly higher than that in WL and FL (*P< 0.01*; **Figure 3**). The bud densities of all bud types showed relatively lower in FL ecosystems compared WL and AM. The soil moisture content at the 0 − 10 cm layers in WL was significantly higher than that in FL and AM, while total C at the 0 − 10 cm layers and total N at 0 − 10 cm layers in AM were markedly higher than that in WL and FL (*P< 0.05*; **Table 1**).

**Table 1:** One-way ANOVA of soil characteristics, vegetation characteristics, and bud bank density of different bud types in wetland (WL), farmland (FL), and Alpine meadow (AM) ecosystems.

Soil characteristics	FL	WL	AM	F	*P*
Soil moisture content (0 − 10cm) (%)	15.458 ± 1.442	36.529 ± 2.764	35.899 ± 2.907	27.634	.000
Total carbon (0 − 10cm) (g/kg)	1.634 ± 0.123	1.605 ± 0.051	3.252 ± 0.351	22.928	.000
Total nitrogen (0 − 10cm) (g/kg)	0.223 ± 0.043	0.159 ± 0.006	0.304 ± 0.028	4.622	.014
Vegetation characteristics	FL	WL	AM	F	P
Vegetation density (No./m^2)	407.000 ± 32.157	943.272 ± 151.363	544.941 ± 32.335	9.114	.000
Shannon-Weiner diversity index	1.234 ± 0.080	0.894 ± 0.118	1.055 ± 0.031	3.863	.026
Aboveground biomass (g)	123.371 ± 9.591	218.081 ± 57.535	93.882 ± 8.887	3.229	.047
Bud density	FL	WL	AM	F	P
Total bud (No./m^2)	149.333 ± 21.490	1514.727 ± 296.666	573.647 ± 91.518	15.313	.000
Rhizome bud (No./m^2)	33.666 ± 10.959	173.818 ± 53.100	220.235 ± 53.516	5.626	.006
Tiller bud (No./m^2)	24.666 ± 8.504	1229.090 ± 279.002	204.235 ± 50.550	15.207	.000
Root sprouting bud (No./m^2)	91.000 ± 19.091	98.181 ± 58.992	149.294 ± 46.496	0.475	.642

The samples were 22 for AM, 24 for WL, and 17 for FL ecosystems. Values are mean ± SE, and the difference was considered significant if *P<* 0.05.

### Effects of vegetation attributes and soil properties on bud banks

**Figure 4:**
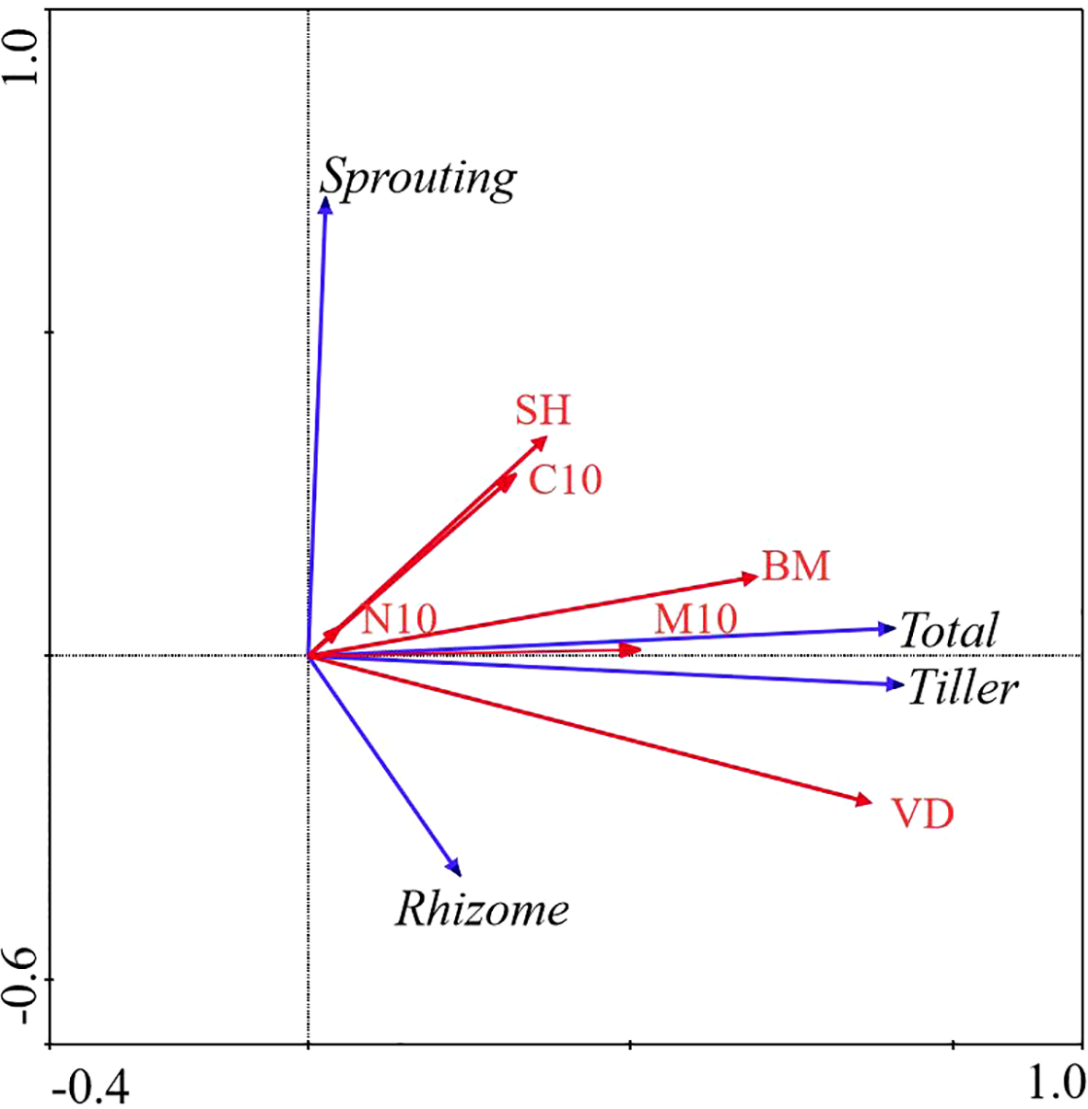
Redundancy Analysis (RDA) of the relationship between bud bank density of different bud types and environmental factors in WL ecosystem. M10, soil moisture content (0 − 10cm) (%); C10, total carbon (0 − 10cm) (g/kg); N10, total nitrogen (0 − 10cm) (g/kg); VD, vegetation density (No./m^2); SH, Shannon-Weiner diversity index; BM, aboveground biomass (g); Total, total bud density; rhizome, rhizome bud density; Tiller, tiller bud density; Sprouting, root sprouting bud density.

In WL ecosystems, all factors combined explained 83.2% of the total variation in bud banks. The soil water content at the 0 − 10 cm layer, vegetation density, and aboveground biomass were significantly correlated with bud banks (*P< 0.05*). Vegetation density was the most critical factor affecting bud banks, explaining 62.2% of the variation of bud banks. Moreover, soil water content at the 0 – 10 cm layer explained 11.9% of the variation of bud banks, whereas the total C at the 0 − 10 cm layer, total N at the 0 − 10 cm layer, and plant diversity had no significant effect on bud banks. The contribution of vegetation attributes and soil properties to the variation in bud bank were 59.1%, 84.5%, and 15.5%, respectively (**Table 2**; **Figure 4**).

**Table 2:** Explanations and contributions of impact factors to the total variation in bud bank for wetland (WL), farmland (FL), and Alpine meadow (AM) ecosystems.

	Controlling Factors	Parameters	Explanations (%)	Contributions (%)	F	*P*
WL	Environment	**M10**	11.9**	**14.3**	**8.766**	**0.006**
		C10	0.3	0.36	0.654	0.494
		N10	0.7	0.84	0.246	0.766
	Vegetation	**VD**	62.2**	**74.7**	**32.943**	**0.002**
		**BM**	5.8*	**6.9**	**5.264**	**0.048**
		SH	2.2	2.6	2.131	0.138
		Total	83.2	100		
FL	Environment	**N10**	12.6*	**35.8**	**3.332**	**0.040**
		C10	8.3	23.6	1.998	0.140
		M10	3.2	9.1	0.871	0.400
	Vegetation	VD	5.8	16.5	1.573	0.212
		BM	5.2	14.8	1.441	0.248
		SH	0.1	0.2	0.021	0.992
		Total	35.1	100		
AM	Environment	**M10**	28.3**	**43.8**	**6.971**	**0.004**
		**C10**	14.8*	**22.9**	**2.641**	**0.070**
		N10	2.4	3.7	0.717	0.572
	Vegetation	VD	3.3	5.1	1.037	0.374
		BM	0.7	1.0	0.193	0.894
		**SH**	15.0*	**23.2**	**4.651**	**0.010**
		Total	64.5	100		

M10, soil moisture content (0 − 10cm) (%); C10, total carbon (C, 0 − 10cm) (g/kg); N10, total nitrogen (0 − 10cm) (g/kg); VD, vegetation density (No./m^2); SH, Shannon-Weiner diversity index; BM, aboveground biomass (g). Values are bold if significant.

* *P*< 0.05. ** *P*< 0.01.

**Figure 5:**
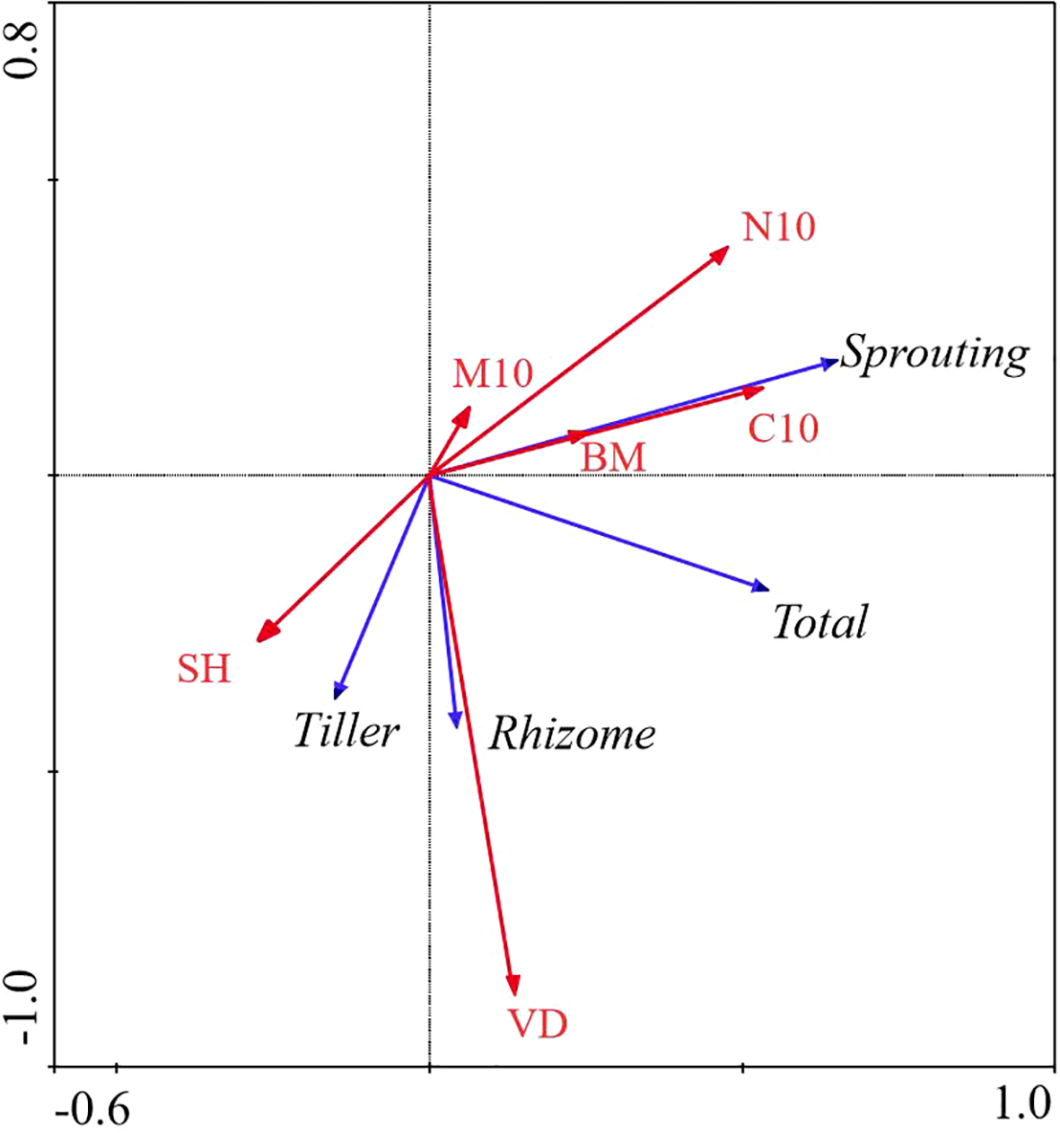
Redundancy Analysis (RDA) of the relationship between bud bank density of different bud types and environmental factors in FL ecosystem. M10, soil moisture content (0 − 10cm) (%); C10, total carbon (0 − 10cm) (g/kg); N10, total nitrogen (0 − 10cm) (g/kg); VD, vegetation density (No./m^2); SH, Shannon-Weiner diversity index; BM, aboveground biomass (g); Total, total bud density; rhizome, rhizome bud density; Tiller, tiller bud density; Sprouting, root sprouting bud density.

All factors in the FL ecosystem explained 35.1% of the total variation in the bud bank. The total N at the 0 − 10 cm layer was significantly correlated with the bud bank (*P< 0.0*5), and it was the decisive factor in affecting the bud bank, explaining 12.6% of the variation of the bud bank. The contribution of vegetation attributes and soil properties to the variation in bud bank was 31.5% and 68.5%, respectively (**Table 2**; **Figure 5**).

**Figure 6:**
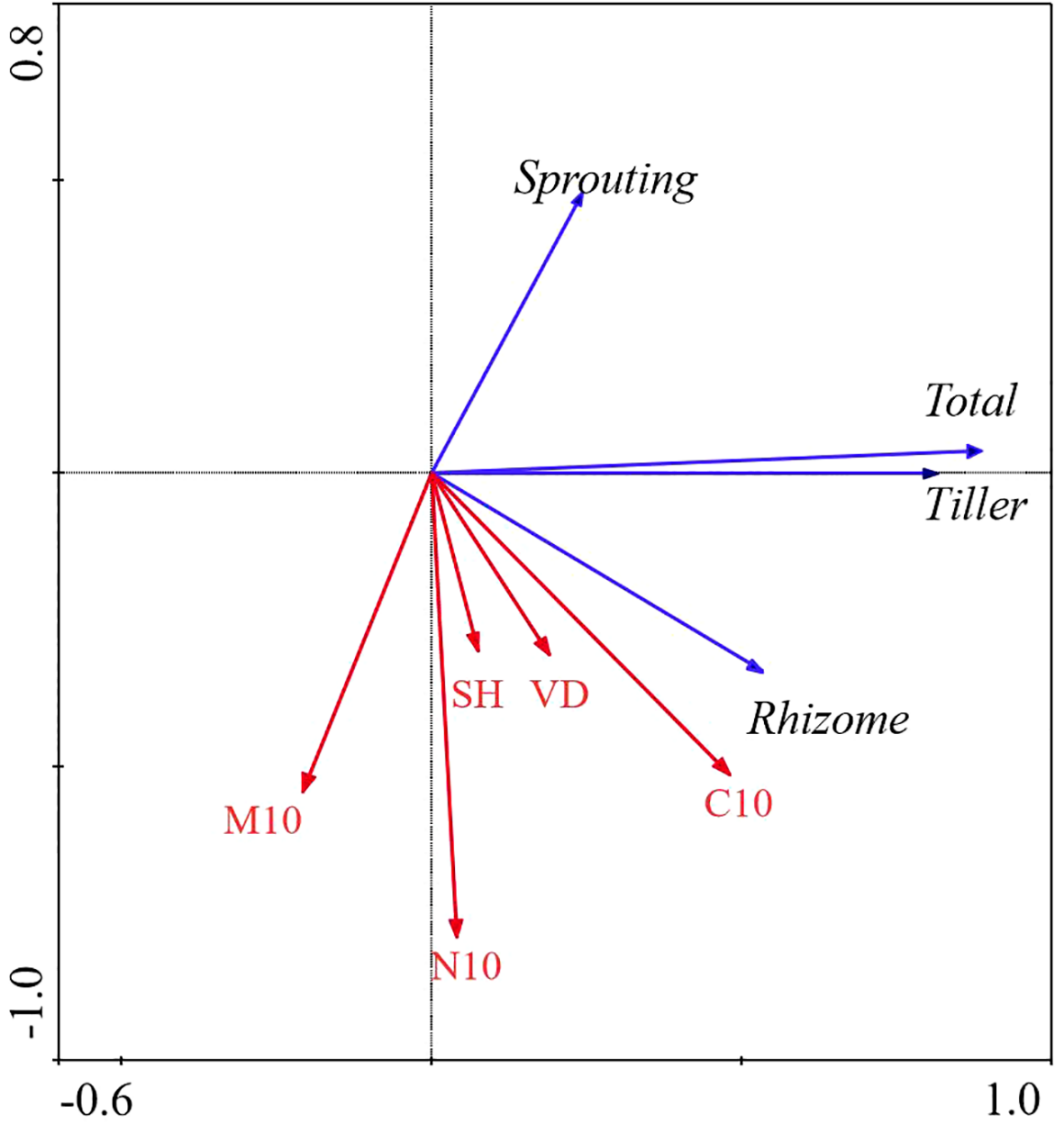
Redundancy Analysis (RDA) of the relationship between bud bank density of different bud types and environmental factors in AM ecosystem. M10, soil moisture content (0 − 10cm) (%); C10, total carbon (0 − 10cm) (g/kg); N10, total nitrogen (0 −10cm) (g/kg); VD, vegetation density (No./m^2); SH, Shannon-Weiner diversity index; BM, aboveground biomass (g); Total, total bud density; rhizome, rhizome bud density; Tiller, tiller bud density; Sprouting, root sprouting bud density.

All factors in the AM ecosystem explained 64.5% of the total variation in the bud bank. The soil water content at the 0 − 10 cm layer, total C at the 0 − 10 cm layer, and plant diversity were significantly correlated with bud banks (*P< 0.0*5). The soil water content at the 0 − 10 cm layer was the most critical factor affecting bud banks, explaining 28.3% of the variation of bud bank, followed by total C at the 0 − 10cm layer, plant diversity explained 14.8% and 15.0% of the variation of bud bank, respectively. The contribution of vegetation attributes and soil properties to the variation in bud bank were 29.6% and 70.4%, respectively (**Table 2**; **Figure 6**).

### Effect of aboveground vegetation and soil properties on bud bank densities of different types

In the WL ecosystem, the densities of total bud and tiller bud were significantly positively correlated with the soil water content (0 − 10 cm layer), vegetation density, and aboveground biomass (*P< 0.05*), and vegetation density was the most critical factor to explain the density variation of total bud and tiller bud. Still, the rhizome bud and root sprouting bud density had no significant relationship with all factors (**Table 3**; **Figure 4**).

**Table 3:** Explanations and contributions of impact factors to the total variation in bank density of different wetland types (WL).

Bud type	Controlling Factors	Parameters	Explanations (%)	Contributions (%)	F	*P*
Rhizome	Environment	C10	10.5†	20.7	3.380	0.080
		M10	13.2†	26.1	3.475	0.090
		N10	14.7†	29.1	3.441	0.084
	Vegetation	VD	5.9	11.6	1.675	0.224
		BM	5.9	11.6	1.602	0.214
		SH	0.3	0.5	0.105	0.746
		Total	50.6	100		
Tiller	Environment	**M10**	12.5**	**14.6**	**11.001**	**0.004**
		C10	0.10	0.1	0.084	0.774
		N10	0.40	0.4	0.493	0.518
	Vegetation	**VD**	65.8**	**76.7**	**38.493**	**0.002**
		**BM**	6.6*	**7.6**	**7.812**	**0.016**
		SH	0.4	0.4	0.482	0.548
		Total	85.8	100		
Root sprouting	Environment	**M10**	**9.8†**	**17.7**	**3.075**	**0.092**
		C10	5.7	10.2	0.282	0.574
		N10	3.6	6.4	1.097	0.326
	Vegetation	VD	13.4†	24.2	3.217	0.082
		BM	15.2†	27.4	4.304	0.070
		SH	7.7	13.8	1.669	0.256
		Total	55.4	100		
Total	Environment	**M10**	13.7*	**17.0**	**8.490**	**0.014**
		C10	0.1	0.1	0.091	0.774
		N10	0.5	0.5	0.407	0.538
	Vegetation	**VD**	55.8**	**69.3**	**25.244**	**0.002**
		**BM**	7.7*	**9.5**	**0.031**	**0.040**
		SH	2.7	3.4	2.292	0.134
		Total	80.5	100		

M10, soil moisture content (0 − 10cm) (%); C10, total carbon (0 − 10cm) (g/kg); N10, total nitrogen (0 − 10cm) (g/kg); VD, vegetation density (No./m^2); SH, Shannon-Weiner diversity index; BM, aboveground biomass (g). Values are bold if significant. * *P*< 0.05. ** *P*< 0.01. † *P*< 0.10.

In the FL ecosystem, the density of the total bud was significantly positively correlated with the total N at the 0 − 10 cm layer, and the density of the root sprouting bud was significantly positively correlated with the total C at the 0 − 10 cm layer. However, rhizome bud and tiller bud density had no significant relationship with all factors (**Table 4**; **Figure 5**).

**Table 4:** Explanations and contributions of impact factors to the total variation inbank density of different types of farmland (FL).

Bud type	Controlling Factors	Parameters	Explanations (%)	Contributions (%)	F	*P*
Rhizome	Environment	C10	0.3	1.5	0.057	0.826
		M10	5.0	26.0	0.115	0.754
		N10	<0.1	<0.1	0.002	0.976
	Vegetation	VD	12.0†	62.5	3.004	0.086
		BM	5.6	29.1	1.426	0.232
		SH	0.8	4.1	0.195	0.626
		Total	19.2	100		
Tiller	Environment	M10	0.80	4.3	0.194	0.636
		C10	0.4	2.2	1.000	0.318
		N10	0.3	1.6	0.075	0.780
	Vegetation	VD	12.7†	69.0	3.213	0.081
		BM	4.0	21.7	0.103	0.738
		SH	0.1	0.5	0.018	0.896
		Total	18.4	100		
Root sprouting	Environment	M10	9.5	20.5	3.187	0.102
		**C10**	14.2*	**30.8**	**3.651**	**0.042**
		N10	10.0†	21.5	2.785	0.088
	Vegetation	VD	10.6†	22.9	3.272	0.092
		BM	1.90	4.1	0.570	0.440
		SH	<0.1	<0.1	0.008	0.912
		Total	46.3	100		
Total	Environment	M10	6.6	19.8	1.776	0.215
		C10	6.2	18.7	1.450	0.230
		**N10**	13.4*	**40.4**	**3.503**	**0.044**
	Vegetation	VD	2.5	7.5	0.674	0.428
		BM	4.2	12.7	1.136	0.322
		SH	0.4	1.2	0.093	0.774
		Total	33.2	100		

M10, soil moisture content (0 − 10cm) (%); C10, total carbon (0 − 10cm) (g/kg); N10, total nitrogen (0 − 10cm) (g/kg); VD, vegetation density (No./m^2); SH, Shannon-Weiner diversity index; BM, aboveground biomass (g). Values are bold if significant. * *P*< 0.05. † *P*< 0.10.

In the AM ecosystem, the densities of total bud and tiller bud were significantly positively correlated with the soil water content (0 − 10 cm layer) and vegetation density (*P< 0.05*), and the soil water content at the 0 − 10 cm layer was the most critical factor that explains the density variation of total bud and tiller bud. The rhizome bud density was significantly positively correlated with total C at the 0 −10 cm layer (*P< 0.05*). Still, the density of root sprouting bud had no significant relationship with all factors (**Table 5**; **Figure 6**).

**Table 5:** Explanations and contributions of impact factors to the total variation in bank density of different types for Alpine meadow (AM).

Bud type	Controlling Factors	Parameters	Explanations (%)	Contributions (%)	F	*P*
Rhizome	Environment	**C10**	25.0*	**54.74.990**		**0.042**
		M10	8.5	18.6	3.246	0.170
		N10	5.7	12.5	1.168	0.278
	Vegetation	VD	1.5	3.2	0.276	0.628
		BM	2.6	5.7	0.512	0.166
		SH	2.5	5.5	0.513	0.438
		Total	45.7	100		
Tiller	Environment	**M10**	25.7*	**35.3**	**6.996**	**0.016**
		C10	9.6	13.2	1.825	0.184
		N10	0.1	0.13	0.059	0.794
	Vegetation	VD	16.9	23.2	3.053	0.126
		BM	0.1	0.14	0.052	0.838
		**SH**	20.3*	**27.9**	**8.848**	**0.020**
		Total	72.8	100		
Root sprouting	Environment	M10	6.9	22.3	1.118	0.280
		C10	9.7	31.3	1.634	0.204
		N10	4.4	14.2	0.714	0.396
	Vegetation	VD	8.1	26.1	1.340	0.256
		BM	1.8	0.1	0.287	0.610
		SH	<0.1	<0.1	0.003	0.968
		Total	31.0	100		
Total	Environment	**M10**	39.4**	**51.1**	**12.706**	**0.004**
		C10	17.2†	22.3	3.122	0.100
		N10	<0.1	<0.1	0.051	0.546
	Vegetation	VD	0.40	0.50	0.189	0.668
		BM	<0.1	<0.1	0.101	0.940
		**SH**	20.1**	**26.1**	**11.179**	**0.008**
		Total	77.1	100		

M10, soil moisture content (0 − 10cm) (%); C10, total carbon (0 − 10cm) (g/kg); N10, total nitrogen (0 − 10cm) (g/kg); VD, vegetation density (No./m^2); SH, Shannon-Weiner diversity index; BM, aboveground biomass (g). Values are bold if significant. * *P*< 0.05. ** *P*< 0.01. † *P*< 0.10.

## Discussion

### Land use change alters the characteristics of bud bank composition and size

Land use change significantly altered the total bud bank density and the bud densities of all types (rhizome bud, tiller bud, and root sprouting bud) across all ecosystems; however, such impacts were higher in WL and AM than in the FL ecosystems. While total bud density, tiller buds, and moisture content were significantly higher in wetland ecosystems, bud densities of all bud types were relatively lower in FL. These results are consistent with previous findings that human-caused disturbances have remarkable adverse effects on belowground bud banks, which have substantial implications on bud regrowth, productivity, and ecological succession (Dalgleish and Hartnett, 2009; Collins and Calabrese, 2012; Deng et al., 2014; Chen et al., 2020). Similarly, results provide empirical evidence that various ecosystems differ in their responses to land use effects, suggesting that variation in species composition and soil characteristics across diverse terrestrial ecosystems underlie their differential responses during ecological perturbation, as observed by previous studies (Lavorel et al., 1997; Boer and Stafford Smith, 2003).

The differential responses of the studied ecosystems (i.e., WL, FL, and AM) can be explained on the following account: firstly, tiller buds in WL accounted for approximately 80% of the total bud densities because of high levels of soil moisture content, total C, and total N at the top 0 −10 cm layer, facilitating plant establishment and population reproduction (Li et al., 2014; Adomako et al., 2020; Ma et al., 2021). These growth and productivity drivers (moisture, total C, and N) primarily promote the build-up of bud density in WL vegetation compared to AM and FL ecosystems (Dalgleish and Hartnett, 2006; Ding et al., 2019). Secondly, higher rhizome and sprouting bud densities in FL and AM compared to WL ecosystems suggest that rhizomes and sprouting buds are highly sensitive to water and nutrient availability (Hiiesalu et al., 2021; Adomako et al., 2022), conditions that are ubiquitously higher in the WL ecosystem and favor tiller buds as grass functional groups represent the dominant populations in wetland vegetation (Williams et al., 2017). Thirdly, FL had relatively the lowest bud densities of all bud types, and this can be attributed to high levels of human-caused disturbances (e.g., grazing, plowing, bush fire, herbicide application) or land use intensification in farmland vegetation compared to low farming activities in wetland zones (Allan et al., 2015). Our results suggest that plant species composition (functional groups) and soil physicochemical parameters determine the resilience and variation in response of ecosystems to anthropogenically mediated disturbances.

### Land use change alters the relative contributions of vegetation attributes and soil properties on bud bank

Vegetation attributes and soil properties strongly correlated with bud banks; however, bud bank demography in the three ecosystems (WL FL, and AM) with different vegetation attributes and soil properties, we found that vegetation density, soil moisture content, and total N was the most critical factors in WL, AM, and FL ecosystems, explaining 62.2, 28.3 and 12.6% accordingly. Higher resource (i.e., soil moisture, total N, and C) availability or growth drivers promoted plant growth, biomass accumulation, and increased vegetation cover and stability (Adomako et al., 2021; Liu et al., 2021; Ma et al., 2021). It is, therefore, plausible to suggest that higher levels of these growth resources invariably increase the vegetation density of the wetland ecosystems, which can also be a function of a greater abundance of belowground bud banks (Liu et al., 2021; Ma et al., 2021), particularly tiller buds that accounted for about 81.14%. Previous studies have indicated that belowground bud banks positively correlate with the net aboveground primary productivity (Qian et al., 2021; Wu and Yu, 2022). Notably, results suggest that wetlands’ maintenance, stability, and productivity are tightly linked with a balance of soil characteristics, bud type, and vegetation attributes (Ma et al., 2021).

Furthermore, studies have indicated that soil moisture is an essential driving force for vegetation succession in the alpine meadow (Heisler-White et al., 2008; An et al., 2019; Zhang et al., 2020; Zhang et al., 2022). In the present study, our analysis indicated that soil water content at the top 0 − 10 cm soil layer significantly influences bud banks, explaining 28.3% of variation of bud banks in the alpine ecosystem. Consistent with most previous studies that reported similar patterns at the top 0 − 10 cm of the soil layer, our results suggest that hydrological regimes can potentially modulate and constrain plant growth, community structure, and stability of alpine meadow ecosystems (Heisler-White et al., 2008; An et al., 2019; Ren et al., 2023), as soil moisture is critical for resprouting and growth of belowground bud banks of all bud types. Additionally, total C, plant diversity, and soil moisture significantly correlated with bud banks, indicating that vegetation and soil physicochemical attributes play crucial roles in ecological succession and productivity output of meadow ecosystems (Hong et al., 2012; Xie et al., 2018; Świerszcz et al., 2019; Plue et al., 2021).

Lastly, total N strongly correlated with and was the most crucial factor influencing bud banks, explaining 12.6% of bud bank variation in farmlands and provides empirical evidence of the extent and magnitude of human-derived disturbances via nutrient enrichments in agrosystems (Isbell et al., 2013; Ren et al., 2019; Adomako et al., 2022). Although N limitation substantially limits plant growth (De Tezanos Pinto and Litchman, 2010; Bracken et al., 2015; Fay et al., 2015; Du et al., 2020; Adomako et al., 2022) and increased fertilization aimed at increasing agricultural output in agrosystems may promote the proliferation of bud banks in short-term period (Liu et al., 2021; Qian et al., 2021; Adomako and Yu, 2023), the long-term effects of N influxes in farmland ecosystems can trigger land degradation (Hamilton et al., 2020; Qian et al., 2021; Owusu et al., 2024). Notably, the FL ecosystem had the least attributes of all bud types measured, which can likely be linked to land use intensification. Our results confirm previous and current findings that N enrichment disrupts plant community composition and plant-microbial interactions, promoting loss of global biodiversity and soil multifunctionality (Galloway et al., 2008; Qian et al., 2021).

### Mechanisms underlying factors affecting bud banks of wetland, alpine meadow, and farmland ecosystems

Overall, soil properties significantly influenced root sprouting and rhizome buds of FL and AM, consistent with previous studies in dune ecosystems (Wu et al., 2021). The measured soil properties (soil moisture, total C and N) represent important factors that plays a pivotal role in many ecosystem processes, such as organic matter mineralization, litter decomposition, and biogeochemical cyclings (Wu et al., 2020; Inoue et al., 2022), which influence nutrient availability and the spatial distribution of resprouting buds and rhizome buds (Xie et al., 2018; Xiao et al., 2021). For example, in a recent study, Xiao et al. (2021) reported a significant influence of soil properties on the spatial distribution of Moso bamboo rhizomes.

Total buds and tiller buds were more strongly correlated with soil qualities in FL and AM ecosystems than aboveground vegetation parameters in WL ecosystems. Strong aboveground recruitment and productivity are tightly linked with the abundance of belowground bud banks, especially tiller buds. Tiller buds constitute hemicryptophytes, and buds emanate from the shoot bases of the mother plant and are protected by leaf sheaths. Therefore, connected tiller buds can receive substantial growth resources from the connected mother plant, facilitating its expansion within plant communities (Ott and Hartnett, 2012). Conversely, in geophytes, root-sprouting and rhizome buds are primarily initiated from subterranean roots and rhizomes (Ott and Hartnett, 2012). These belowground structures are sensitive to water and nutrient availability in their ambient environment (Passioura, 1988; Wu et al., 2020). Therefore, bud banks can be a major driving force limiting productivity in wetland ecosystems. Unlike in FL and AM ecosystems, many wetland species are adapted to modifications in soil conditions and human-caused disturbances resulting from land use intensification. Thus, tiller buds connected to parent plants can obtain parental resource nourishments and withstand land use pressures in their ecosystem (Ott and Hartnett, 2012; Wu et al., 2020).

In contrast, as rhizome buds and root sprouting buds mainly initiate from underground roots and rhizomes, they could directly forage water or nutrients in the surrounding soil (Vesk and Westoby, 2004; Deng et al., 2013). Previous studies demonstrated that plants tend to produce more rhizome buds to increase foraging for favorable patches for persistence and regeneration in a resource-poor region, while in a relatively low resources environment, more tiller buds are produced to increase dominance and resource capture (Qian et al., 2017; Wu et al., 2021). Our study further illustrates that root sprouting buds are related to the soil nutrient content of the top (0 − 10 cm) layer because of direct roots sensitivity to soil nutrients (Klimeš and Klimešová, 1999; Ma et al., 2019).

## Conclusions

Exploring belowground bud bank responses in different ecosystems is essential for understanding the adaptive strategies and vegetation restoration under the ongoing land use changes. We found that land-use change alters bud bank composition and size characteristics and alters the relative contributions of vegetation attributes and soil properties on bud banks. In WL environments, vegetation density is a crucial determinant; soil conditions are the most important factor affecting bud banks in FL and AM habitats. For different bud types, total buds and tiller buds rely more on vegetation density in WL ecosystems, but total buds and tiller buds are more related to soil properties in ecosystems of FL and AM. Rhizome and root sprouting buds could buffer vegetation restoration under land use change. Results indicate that vegetation and soil attributes play critical roles, underly the differential responses and the composition of bud banks of different ecosystems. Given the predicted climate change impacts and rapid expansion of industrialization and settlements, similar studies involving more climate change factors under varying climatic conditions may be highly informative and insightful.

## Data availability statement

The raw data supporting the conclusions of this article will be made available by the authors, without undue reservation.

## Author contributions

JW: Conceptualization, Funding acquisition, Investigation, Methodology, Writing – original draft. XH: Investigation, Writing – original draft. LX: Methodology, Writing – original draft. QZ: Methodology, Writing – original draft. YW: Investigation, Writing – original draft. ZG: Writing – review & editing. MOA: Writing – review & editing. QM: Writing – review & editing.

## References

[B1] Adomako M. O. Xue W. Du D.-L. Yu F.-H. (2021). Soil biota and soil substrates influence responses of the rhizomatous clonal grass *Leymus chinensis* to nutrient heterogeneity. Plant Soil 465, 19–29. doi: 10.1007/s11104-021-04967-0

[B2] Adomako M. O. Xue W. Du D.-L. Yu F.-H. (2022). Soil microbe-mediated N:P stoichiometric effects on *Solidago canadensis* performance depend on nutrient levels. Microbial Ecol. 83, 960–970. doi: 10.1007/s00248-021-01814-8 34279696

[B3] Adomako M. O. Xue W. Tang M. Du D.-L. Yu F.-H. (2020). Synergistic effects of soil microbes on *Solidago canadensis* depend on water and nutrient availability. Microbial Ecol. 80, 837–845. doi: 10.1007/s00248-020-01537-2 32561944

[B4] Adomako M. O. Yu F.-H. (2023). Functional group dominance-mediated N:P effects on community productivity depend on soil nutrient levels. Rhizosphere 26, 100692. doi: 10.1016/j.rhisph.2023.100692

[B5] Allan E. Manning P. Alt F. Binkenstein J. Blaser S. Blüthgen N.. (2015). Land use intensification alters ecosystem multifunctionality via loss of biodiversity and changes to functional composition. Ecol. Lett. 18, 834–843. doi: 10.1111/ele.12469 26096863 PMC4744976

[B6] An Y. Gao Y. Liu X. H. Tong S. Z. (2019). Interactions of soil moisture and plant community properties in meadows restored from abandoned farmlands on the Sanjiang Plain, China. Community Ecol. 20, 20–27. doi: 10.1556/168.2019.20.1.3

[B7] Boer M. Stafford Smith M. (2003). A plant functional approach to the prediction of changes in Australian rangeland vegetation under grazing and fire. J. Vegetation Sci. 14, 333–344. doi: 10.1111/j.1654-1103.2003.tb02159.x

[B8] Bracken M. E. S. Hillebrand H. Borer E. T. Seabloom E. W. Cebrian J. Cleland E. E.. (2015). Signatures of nutrient limitation and co-limitation: responses of autotroph internal nutrient concentrations to nitrogen and phosphorus additions. Oikos 124, 113–121. doi: 10.1111/oik.01215

[B9] Chang C. C. Turner B. L. (2019). Ecological succession in a changing world. J. Ecol. 107, 503–509. doi: 10.1111/1365-2745.13132

[B10] Chen X. Zhu L. Deng Z. Xie Y. Chen X. Li F.. (2020). Effects of clipping on bud bank and population regeneration of *Triarrhena lutarioriparia* in Dongting Lake wetland, China. Wetlands 40, 2635–2642. doi: 10.1007/s13157-020-01311-7

[B11] Clarke P. J. Lawes M. J. Midgley J. J. Lamont B. B. Ojeda F. Burrows G. E.. (2013). Resprouting as a key functional trait: how buds, protection and resources drive persistence after fire. New Phytol. 197, 19–35. doi: 10.1111/nph.12001 23110592

[B12] Collins S. L. Calabrese L. B. (2012). Effects of fire, grazing and topographic variation on vegetation structure in tallgrass prairie. J. Vegetation Sci. 23, 563–575. doi: 10.1111/j.1654-1103.2011.01369.x

[B13] Dalgleish H. J. Hartnett D. C. (2006). Below-ground bud banks increase along a precipitation gradient of the North American Great Plains: a test of the meristem limitation hypothesis. New Phytol. 171, 81–89. doi: 10.1111/j.1469-8137.2006.01739.x 16771984

[B14] Dalgleish H. J. Hartnett D. C. (2009). The effects of fire frequency and grazing on tallgrass prairie productivity and plant composition are mediated through bud bank demography. Plant Ecol. 201, 411–420. doi: 10.1007/s11258-008-9562-3

[B15] Deng L. Sweeney S. Shangguan Z. P. (2014). Grassland responses to grazing disturbance: plant diversity changes with grazing intensity in a desert steppe. Grass Forage Sci. 69, 524–533. doi: 10.1111/gfs.12065

[B16] Deng Z. M. Chen X. S. Xie Y. H. Li X. Pan Y. Li F. (2013). Effects of size and vertical distribution of buds on sprouting and plant growth of the clonal emergent macrophyte Miscanthus sacchariflorus (Poaceae). Aquat Bot. 104, 12–14. doi: 10.1016/j.aquabot.2012.08.004

[B17] De Tezanos Pinto P. Litchman E. (2010). Interactive effects of N:P ratios and light on nitrogen-fixer abundance. Oikos 119, 567–575. doi: 10.1111/j.1600-0706.2009.17924.x

[B18] Ding X. Su P. Zhou Z. Shi R. (2019). Belowground bud bank distribution and aboveground community characteristics along different moisture gradients of alpine meadow in the Zoige Plateau, China. Sustainability 11, 2602. doi: 10.3390/su11092602

[B19] Du E. Terrer C. Pellegrini A. F. A. Ahlström A. Van Lissa C. J. Zhao X.. (2020). Global patterns of terrestrial nitrogen and phosphorus limitation. Nat. Geosci. 13, 221–226. doi: 10.1038/s41561-019-0530-4

[B20] Fay P. A. Prober S. M. Harpole W. S. Knops J. M. H. Bakker J. D. Borer E. T.. (2015). Grassland productivity limited by multiple nutrients. Nat. Plants 1, 15080. doi: 10.1038/nplants.2015.80 27250253

[B21] Galloway J. N. Townsend A. R. Erisman J. W. Bekunda M. Cai Z. Freney J. R.. (2008). Transformation of the nitrogen cycle: Recent trends, questions, and potential solutions. Science 320, 889–892. doi: 10.1126/science.1136674 18487183

[B22] Hamilton D. J. Bulmer R. H. Schwendenmann L. Lundquist C. J. (2020). Nitrogen enrichment increases greenhouse gas emissions from emerged intertidal sandflats. Sci. Rep. 10, 6686. doi: 10.1038/s41598-020-62215-4 32317656 PMC7174373

[B23] Heisler-White J. L. Knapp A. K. Kelly E. F. (2008). Increasing precipitation event size increases aboveground net primary productivity in a semi-arid grassland. Oecologia 158, 129–140. doi: 10.1007/s00442-008-1116-9 18670792

[B24] Hiiesalu I. Klimešová J. Doležal J. Mudrák O. Götzenberger L. Horník J.. (2021). Hidden below-ground plant diversity buffers against species loss during land-use change in species-rich grasslands. J. Vegetation Sci. 32, e12971. doi: 10.1111/jvs.12971

[B25] Hong J. Liu S. Shi G. Zhang Y. (2012). Soil seed bank techniques for restoring wetland vegetation diversity in Yeyahu Wetland, Beijing. Ecol. Eng. 42, 192–202. doi: 10.1016/j.ecoleng.2012.01.004

[B26] Hoover D. L. Knapp A. K. Smith M. D. (2014). Resistance and resilience of a grassland ecosystem to climate extremes. Ecology 95, 2646–2656. doi: 10.1890/13-2186.1

[B27] Hou L. Kong W. Qiu Q. Yao Y. Bao K. Zhang L.. (2022). Dynamics of soil N cycling and its response to vegetation presence in an eroding watershed of the Chinese Loess Plateau. Agriculture Ecosyst. Environ. 336, 108020. doi: 10.1016/j.agee.2022.108020

[B28] Inoue T. Akaji Y. Kohzu A. Hinokidani K. Adachi H. Kezuka M.. (2022). Relationship between plant growth and soil chemical properties in a mangrove afforestation stand, Kiribati. Plant Soil 479, 559–571. doi: 10.1007/s11104-022-05545-8

[B29] Isbell F. Reich P. B. Tilman D. Hobbie S. E. Polasky S. Binder S. (2013). Nutrient enrichment, biodiversity loss, and consequent declines in ecosystem productivity. Proc. Natl. Acad. Sci. 110, 11911–11916. doi: 10.1073/pnas.1310880110 23818582 PMC3718098

[B30] Klimeš L. Klimešová J. (1999). Root sprouting in Rumex acetosella under different nutrient levels. Plant Ecol. 141, 33–39. doi: 10.1023/A:1009877923773

[B31] Klimešová J. Martínková J. Bartušková A. Ott J. P. (2023). Belowground plant traits and their ecosystem functions along aridity gradients in grasslands. Plant Soil 487, 39–48. doi: 10.1007/s11104-023-05964-1

[B32] Lavorel S. Mcintyre S. Landsberg J. Forbes T. D. A. (1997). Plant functional classifications: from general groups to specific groups based on response to disturbance. Trends Ecol. Evol. 12, 474–478. doi: 10.1016/S0169-5347(97)01219-6 21238163

[B33] Li Y.-Y. Dong S.-K. Wen L. Wang X.-X. Wu Y. (2014). Soil carbon and nitrogen pools and their relationship to plant and soil dynamics of degraded and artificially restored grasslands of the Qinghai–Tibetan Plateau. Geoderma 213, 178–184. doi: 10.1016/j.geoderma.2013.08.022

[B34] Li F.-R. Liu J.-L. Ren W. Liu L.-L. (2018). Land-use change alters patterns of soil biodiversity in arid lands of northwestern China. Plant Soil 428, 371–388. doi: 10.1007/s11104-018-3673-y

[B35] Liu H. Wu Y. Xu H. Ai Z. Zhang J. Liu G.. (2021). N enrichment affects the arbuscular mycorrhizal fungi-mediated relationship between a C4 grass and a legume. Plant Physiol. 187, 1519–1533. doi: 10.1093/plphys/kiab328 34618052 PMC8566264

[B36] Luo W. Muraina T. O. Griffin-Nolan R. J. Te N. Qian J. Yu Q.. (2023). High below-ground bud abundance increases ecosystem recovery from drought across arid and semiarid grasslands. J. Ecol 111, 2038–2048. doi: 10.1111/1365-2745.14160

[B37] Ma Q. Qian J. Q. T L. Liu Z. M. (2019). Responses of belowground bud bank to disturbance and stress in the sand dune ecosystem. Ecol Indic. 106, 1–7. doi: 10.1016/j.ecolind.2019.105521

[B38] Ma M. Zhu Y. Wei Y. Zhao N. (2021). Soil nutrient and vegetation diversity patterns of alpine wetlands on the Qinghai-Tibetan Plateau. Sustainability (Elsevier) 13, 6221. doi: 10.3390/su13116221

[B39] Mobilian C. Craft C. (2021). Wetland soils: Physical and chemical properties and biogeochemical processes (Elsevier). doi: 10.1016/B978-0-12-819166-8.00049-9

[B40] Newbold T. Hudson L. N. Hill S. L. L. Contu S. Lysenko I. Senior R. A.. (2015). Global effects of land use on local terrestrial biodiversity. Nature 520, 45–50. doi: 10.1038/nature14324 25832402

[B41] Ott J. P. Hartnett D. C. (2012). Contrasting bud bank dynamics of two co-occurring grasses in tallgrass prairie: implications for grassland dynamics. Plant Ecol. 213, 1437–1448. doi: 10.1007/s11258-012-0102-9

[B42] Ott J. P. Hartnett D. C. (2015). Bud-bank and tiller dynamics of co-occurring C3 caespitose grasses in mixed-grass prairie. Am. J. Bot. 102, 1462–1471. doi: 10.3732/ajb.1500039 26373977

[B43] Ott J. P. Klimešová J. Hartnett D. C. (2019). The ecology and significance of below-ground bud banks in plants. Ann. Bot. 123, 1099–1118. doi: 10.1093/aob/mcz051 31167028 PMC6612937

[B44] Owusu S. M. Adomako M. O. Qiao H. (2024). Organic amendment in climate change mitigation: Challenges in an era of micro- and nanoplastics. Sci. Total Environ. 907, 168035. doi: 10.1016/j.scitotenv.2023.168035 37907110

[B45] Passioura J. B. (1988). Water transport in and to roots. Annu. Rev. Plant Physiol. Plant Mol. Biol. 39, 245–265. doi: 10.1146/annurev.pp.39.060188.001333

[B46] Peng J. Li Y. Tian L. Liu Y. Wang Y. (2015). Vegetation dynamics and associated driving forces in eastern China during 1999–2008. Remote Sens. 7, 13641–13663. doi: 10.3390/rs71013641

[B47] Plue J. Cousins S. A. O. (2018). Seed dispersal in both space and time is necessary for plant diversity maintenance in fragmented landscapes. Oikos 127, 780–791. doi: 10.1111/oik.04813

[B48] Plue J. Van Calster H. Auestad I. Basto S. Bekker R. M. Bruun H. H.. (2021). Buffering effects of soil seed banks on plant community composition in response to land use and climate. Global Ecol. Biogeography 30, 128–139. doi: 10.1111/geb.13201

[B49] Qian J. Guo Z. Muraina T. O. Te N. Griffin-Nolan R. J. Song L.. (2022). Legacy effects of a multi-year extreme drought on belowground bud banks in rhizomatous vs bunchgrass-dominated grasslands. Oecologia 198, 763–771. doi: 10.1007/s00442-022-05133-8 35230515

[B50] Qian J. Wang Z. Klimešová J. Lü X. Kuang W. Liu Z.. (2017). Differences in below-ground bud bank density and composition along a climatic gradient in the temperate steppe of northern China. Ann. Bot. 120, 755–764. doi: 10.1093/aob/mcx072 28633337 PMC5691867

[B51] Qian J. Wang Z. Klimešová J. Lü X. Zhang C. (2021). Belowground bud bank and its relationship with aboveground vegetation under watering and nitrogen addition in temperate semiarid steppe. Ecol. Indic. 125, 107520. doi: 10.1016/j.ecolind.2021.107520

[B52] Qian J. Zhang Z. Dong Y. Ma Q. Yu Q. Zhu J.. (2023). Responses of bud banks and shoot density to experimental drought along an aridity gradient in temperate grasslands. Funct. Ecol. 37, 1211–1220. doi: 10.1111/1365-2435.14301

[B53] Reddy K. R. Clark M. W. Delaune R. D. Kongchum M. (2013). Physicochemical characterization of wetland soils. Methods Biogeochemistry Wetlands 10, 41–54. doi: 10.2136/sssabookser10.c3

[B54] Ren G. Du Y. Yang B. Wang J. Cui M. Dai Z.. (2023). Influence of precipitation dynamics on plant invasions: response of alligator weed (Alternanthera philoxeroides) and co-occurring native species to varying water availability across plant communities. Biol. Invasions 25, 519–532. doi: 10.1007/s10530-022-02931-2

[B55] Ren G.-Q. Li Q. Li Y. Li J. Opoku Adomako M. Dai Z.-C.. (2019). The enhancement of root biomass increases the competitiveness of an invasive plant against a co-occurring native plant under elevated nitrogen deposition. Flora 261, 151486. doi: 10.1016/j.flora.2019.151486

[B56] Semchenko M. Zobel K. Heinemeyer A. Hutchings M. J. (2008). Foraging for space and avoidance of physical obstructions by plant roots: a comparative study of grasses from contrasting habitats. New Phytol. 179, 1162–1170. doi: 10.1111/j.1469-8137.2008.02543.x 18627492

[B57] Semenchuk P. Plutzar C. Kastner T. Matej S. Bidoglio G. Erb K.-H.. (2022). Relative effects of land conversion and land-use intensity on terrestrial vertebrate diversity. Nat. Commun. 13, 615. doi: 10.1038/s41467-022-28245-4 35105884 PMC8807604

[B58] Simkin R. D. Seto K. C. Mcdonald R. I. Jetz W. (2022). Biodiversity impacts and conservation implications of urban land expansion projected to 2050. Proc. Natl. Acad. Sci. 119, e2117297119. doi: 10.1073/pnas.2117297119 35286193 PMC8944667

[B59] Świerszcz S. Nobis M. Maślak M. Smieja A. Kojs P. Nowak S.. (2019). Varied response of underground and aboveground plant matter: functional diversity of three different vegetational types after translocation to reclaimed postindustrial land. Land Degradation Dev. 30, 2287–2297. doi: 10.1002/ldr.3419

[B60] Vanderweide B. L. Hartnett D. C. (2015). Belowground bud bank response to grazing under severe, short-term drought. Oecologia 178, 795–806. doi: 10.1007/s00442-015-3249-y 25676105

[B61] Vesk P. A. Westoby M. (2004). Funding the bud bank: a review of the costs of buds. Oikos 106, 200–208. doi: 10.1111/j.0030-1299.2004.13204.x

[B62] Williams A. S. Kiniry J. R. Mushet D. Smith L. M. Mcmurry S. Attebury K.. (2017). Model parameters for representative wetland plant functional groups. Ecosphere 8, e01958. doi: 10.1002/ecs2.1958

[B63] Winkler K. Fuchs R. Rounsevell M. Herold M. (2021). Global land use changes are four times greater than previously estimated. Nat. Commun. 12, 2501. doi: 10.1038/s41467-021-22702-2 33976120 PMC8113269

[B64] Wu J. Chen X. Xu L. Qian J. Liu Z. (2021). The spatial pattern of the belowground bud bank and its responses to soil water status in the interdune lowlands of active sand dunes of Inner Mongolia, China. Restor. Ecol. 29, e13223. doi: 10.1111/rec.13223

[B65] Wu J. Wang Y. Ma Q. Liu Z. (2020). Roles of aboveground vegetation, soil properties, and disturbance in determining belowground bud bank in sand dune ecosystems. Environ. Exp. Bot. 178, 104155. doi: 10.1016/j.envexpbot.2020.104155

[B66] Wu J. Yu F.-H. (2022). Belowground bud bank of invasive plants contributes to their successful invasion in coastal wetlands. Restor. Ecol. 31, e13821. doi: 10.1111/rec.13821

[B67] Xiao L. Li C. Cai Y. Zhou T. Zhou M. Gao X.. (2021). Interactions between soil properties and the rhizome-root distribution in a 12-year Moso bamboo reforested region: Combining ground-penetrating radar and soil coring in the field. Sci. Total Environ. 800, 149467. doi: 10.1016/j.scitotenv.2021.149467 34391161

[B68] Xie H. H. Wu Q. G. Hu J. Y. Yu L. F. Bie P. F. Wang H.. (2018). Changes in soil physical and chemical properties during the process of alpine meadow degradation along the Eastern Qinghai-Tibet Plateau. Eurasian Soil Sci. 51, 1440–1446. doi: 10.1134/S1064229318130045

[B69] Xu C. Ke Y. Zhou W. Luo W. Ma W. Song L.. (2021). Resistance and resilience of a semi-arid grassland to multi-year extreme drought. Ecol. Indic. 131, 108139. doi: 10.1016/j.ecolind.2021.108139

[B70] Zhang Q.-P. Fang R.-Y. Deng C.-Y. Zhao H.-J. Shen M.-H. Wang Q. (2022). Slope aspect effects on plant community characteristics and soil properties of alpine meadows on Eastern Qinghai-Tibetan plateau. Ecol. Indic. 143, 109400. doi: 10.1016/j.ecolind.2022.109400

[B71] Zhang J.-T. Mu C.-S. Wang D.-L. Wang J.-F. Chen G.-X. (2009). Shoot population recruitment from a bud bank over two seasons of undisturbed growth of Leymus chinensis. Botany 87, 1242–1249. doi: 10.1139/B09-080

[B72] Zhang W. Yi S. Qin Y. Sun Y. Shangguan D. Meng B.. (2020). Effects of patchiness on surface soil moisture of alpine meadow on the Northeastern Qinghai-Tibetan Plateau: Implications for grassland restoration. Remote Sens. 12, 4121. doi: 10.3390/rs12244121

[B73] Zhang D. M. Zhao W. Z. Luo W. C. (2019). Effect of the population density on belowground bud bank of a rhizomatous clonal plant Leymus secalinus in Mu Us sandy land. J. Plant Res. 132, 69–80. doi: 10.1007/s10265-018-01080-9 30610496

[B74] Zhao G. Dong J. Yang J. Wang H. Dai J. Zhou Y.. (2023). Cropland expansion delays vegetation spring phenology according to satellite and *in-situ* observations. Agriculture Ecosyst. Environ. 356, 108651. doi: 10.1016/j.agee.2023.108651

[B75] Zuo X. Zhao X. Zhao H. Zhang T. Guo Y. Li Y.. (2009). Spatial heterogeneity of soil properties and vegetation–soil relationships following vegetation restoration of mobile dunes in Horqin Sandy Land, Northern China. Plant Soil 318, 153–167. doi: 10.1007/s11104-008-9826-7

